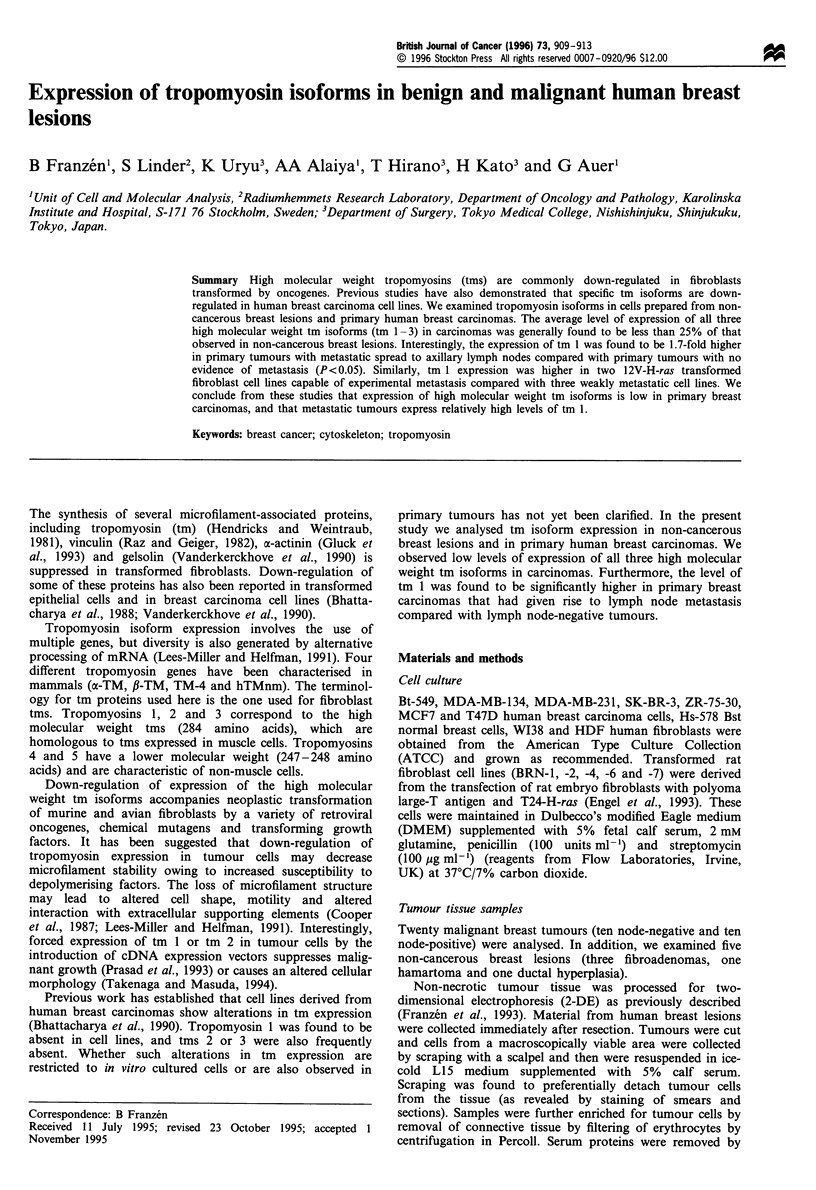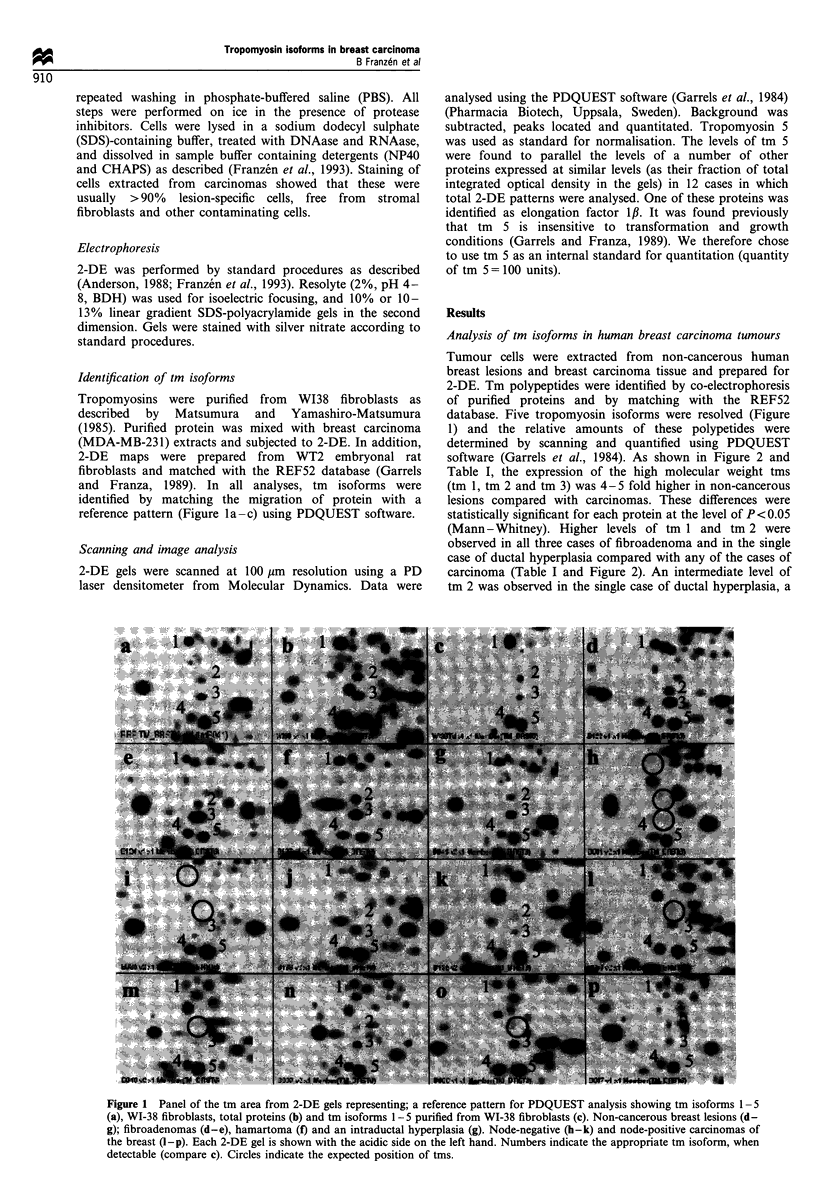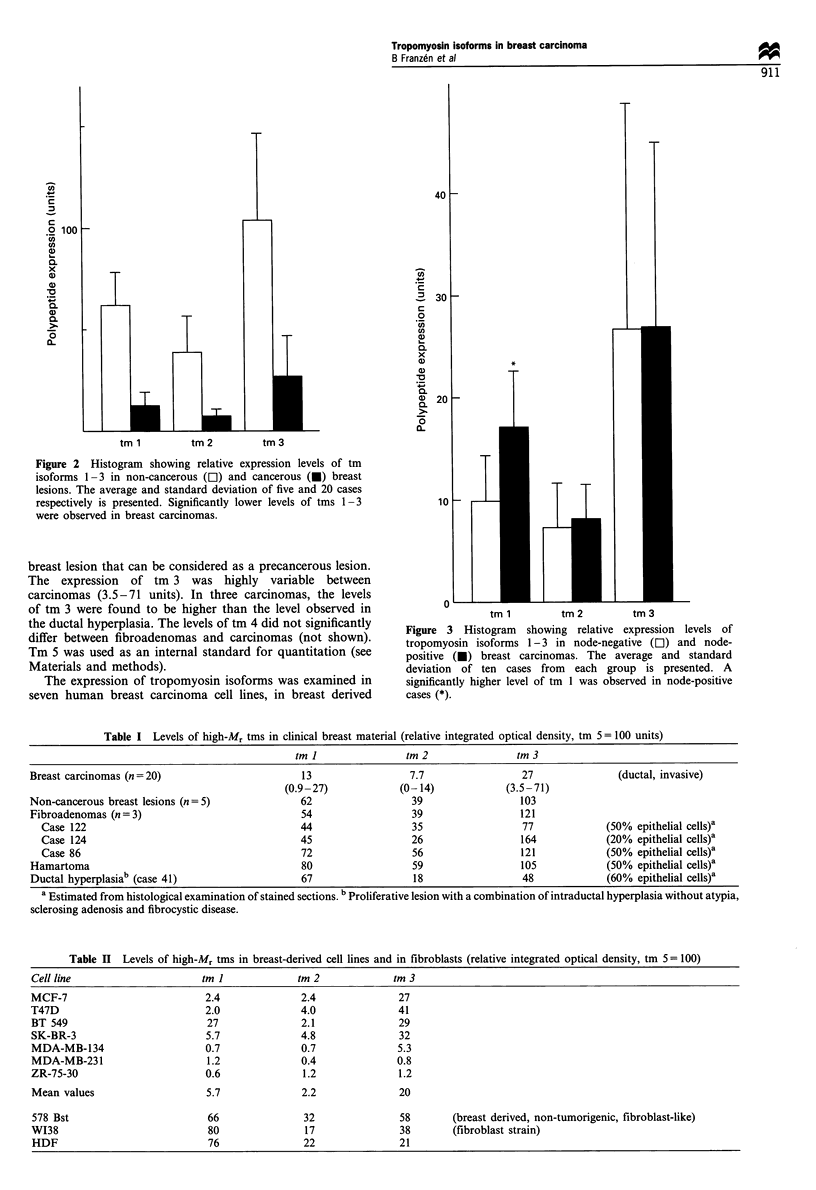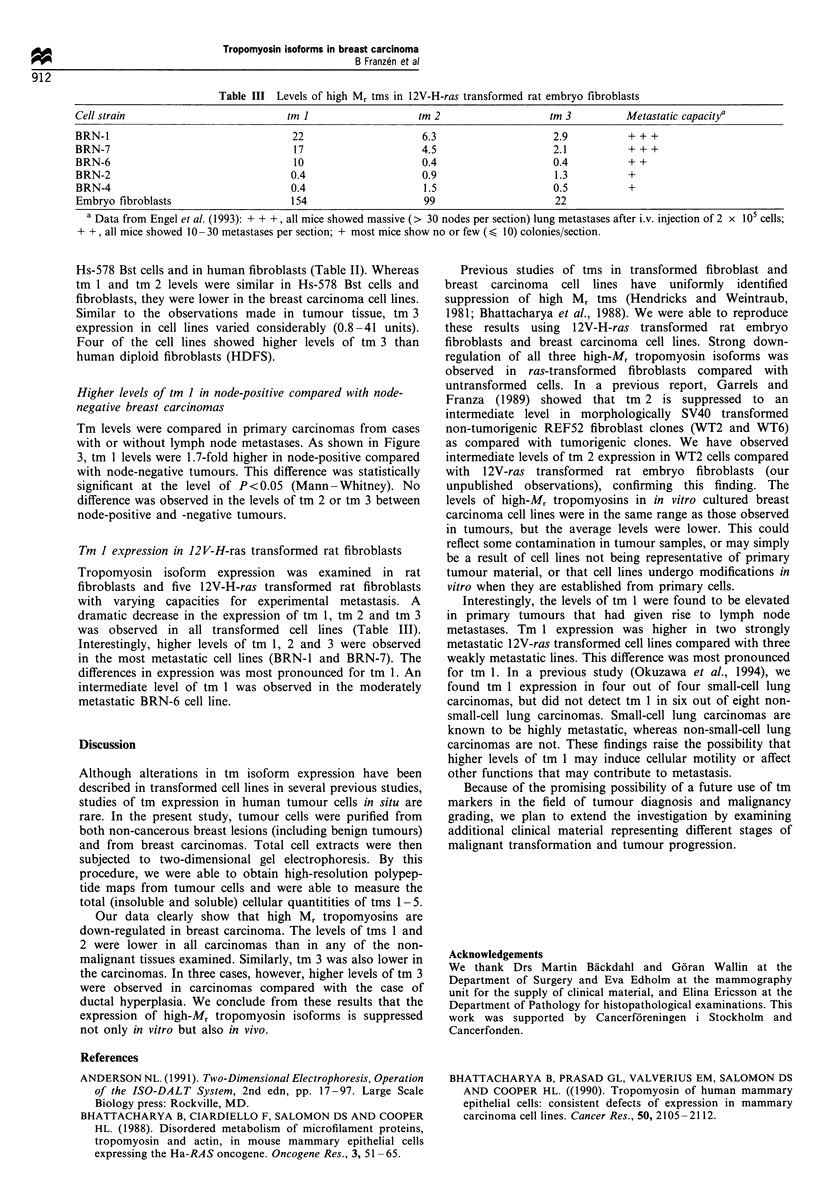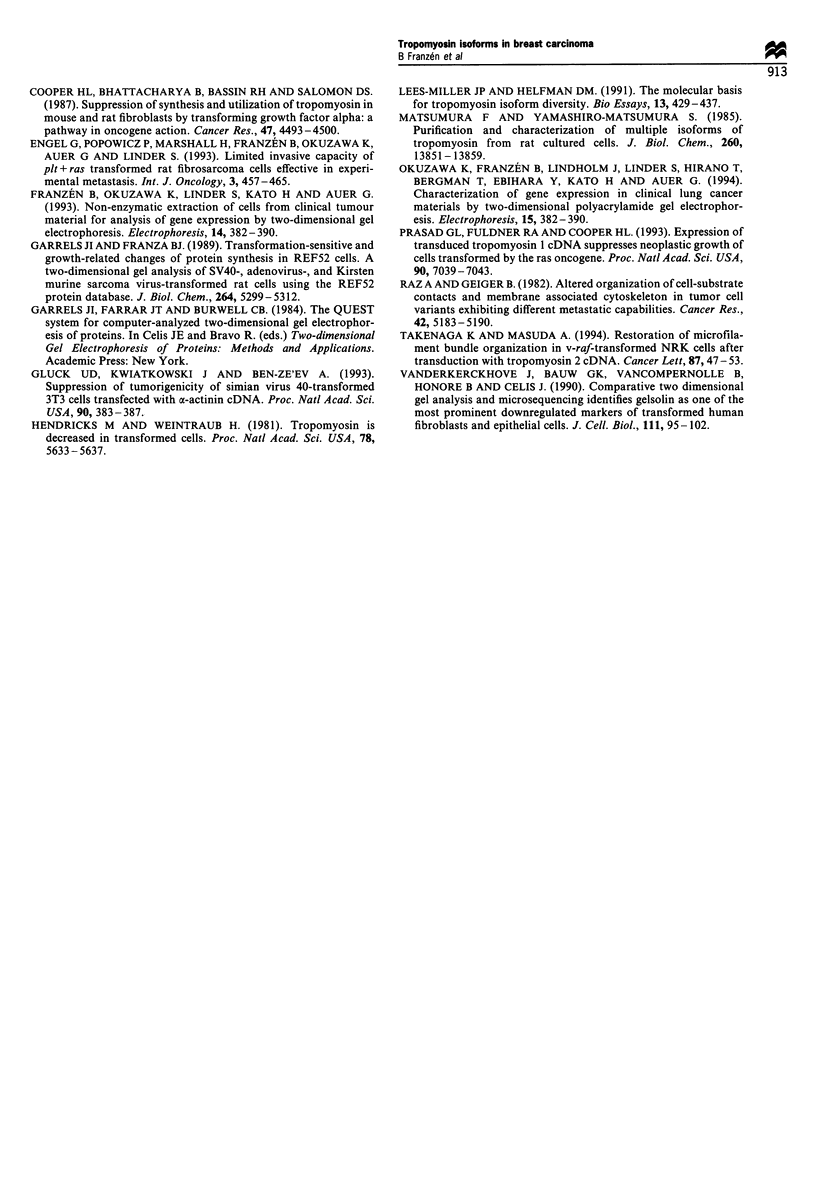# Expression of tropomyosin isoforms in benign and malignant human breast lesions.

**DOI:** 10.1038/bjc.1996.162

**Published:** 1996-04

**Authors:** B. Franzén, S. Linder, K. Uryu, A. A. Alaiya, T. Hirano, H. Kato, G. Auer

**Affiliations:** Unit of Cell and Molecular Analysis, Karolinska Institute and Hospital, Stockholm, Sweden.

## Abstract

**Images:**


British Journal of Cancer (1996) 73, 909-913
© 1996 Stockton Press All rights reserved 0007-0920/96 $12.00

### Expression of tropomyosin isoforms in benign and malignant human breast lesions

B Franzén[1], S Linder[2], K Uryu[3], AA Alaiya[1], T Hirano[3], H Kato[3] and G Auer[1]

[1]*Unit of Cell and Molecular Analysis,* [2]*Radiumhemmets Research Laboratory, Department of Oncology and Pathology, Karolinska Institute and Hospital, S-171 76 Stockholm, Sweden;* [3]*Department of Surgery, Tokyo Medical College, Nishishinjuku, Shinjukuku, Tokyo, Japan.*

**Summary** High molecular weight tropomyosins (tms) are commonly down-regulated in fibroblasts transformed by oncogenes. Previous studies have also demonstrated that specific tm isoforms are down-regulated in human breast carcinoma cell lines. We examined tropomyosin isoforms in cells prepared from non-cancerous breast lesions and primary human breast carcinomas. The average level of expression of all three high molecular weight tm isoforms (tm 1-3) in carcinomas was generally found to be less than 25% of that observed in non-cancerous breast lesions. Interestingly, the expression of tm 1 was found to be 1.7-fold higher in primary tumours with metastatic spread to axillary lymph nodes compared with primary tumours with no evidence of metastasis ($P<0.05$). Similarly, tm 1 expression was higher in two 12V-H-*ras* transformed fibroblast cell lines capable of experimental metastasis compared with three weakly metastatic cell lines. We conclude from these studies that expression of high molecular weight tm isoforms is low in primary breast carcinomas, and that metastatic tumours express relatively high levels of tm 1.

**Keywords:** breast cancer; cytoskeleton; tropomyosin

The synthesis of several microfilament-associated proteins, including tropomyosin (tm) (Hendricks and Weintraub, 1981), vinculin (Raz and Geiger, 1982), α-actinin (Gluck *et al.*, 1993) and gelsolin (Vanderkerckhove *et al.*, 1990) is suppressed in transformed fibroblasts. Down-regulation of some of these proteins has also been reported in transformed epithelial cells and in breast carcinoma cell lines (Bhattacharya *et al.*, 1988; Vanderkerckhove *et al.*, 1990).

Tropomyosin isoform expression involves the use of multiple genes, but diversity is also generated by alternative processing of mRNA (Lees-Miller and Helfman, 1991). Four different tropomyosin genes have been characterised in mammals (α-TM, β-TM, TM-4 and hTMnm). The terminology for tm proteins used here is the one used for fibroblast tms. Tropomyosins 1, 2 and 3 correspond to the high molecular weight tms (284 amino acids), which are homologous to tms expressed in muscle cells. Tropomyosins 4 and 5 have a lower molecular weight (247-248 amino acids) and are characteristic of non-muscle cells.

Down-regulation of expression of the high molecular weight tm isoforms accompanies neoplastic transformation of murine and avian fibroblasts by a variety of retroviral oncogenes, chemical mutagens and transforming growth factors. It has been suggested that down-regulation of tropomyosin expression in tumour cells may decrease microfilament stability owing to increased susceptibility to depolymerising factors. The loss of microfilament structure may lead to altered cell shape, motility and altered interaction with extracellular supporting elements (Cooper *et al.*, 1987; Lees-Miller and Helfman, 1991). Interestingly, forced expression of tm 1 or tm 2 in tumour cells by the introduction of cDNA expression vectors suppresses malignant growth (Prasad *et al.*, 1993) or causes an altered cellular morphology (Takenaga and Masuda, 1994).

Previous work has established that cell lines derived from human breast carcinomas show alterations in tm expression (Bhattacharya *et al.*, 1990). Tropomyosin 1 was found to be absent in cell lines, and tms 2 or 3 were also frequently absent. Whether such alterations in tm expression are restricted to *in vitro* cultured cells or are also observed in primary tumours has not yet been clarified. In the present study we analysed tm isoform expression in non-cancerous breast lesions and in primary human breast carcinomas. We observed low levels of expression of all three high molecular weight tm isoforms in carcinomas. Furthermore, the level of tm 1 was found to be significantly higher in primary breast carcinomas that had given rise to lymph node metastasis compared with lymph node-negative tumours.

### Materials and methods

### *Cell culture*

Bt-549, MDA-MB-134, MDA-MB-231, SK-BR-3, ZR-75-30, MCF7 and T47D human breast carcinoma cells, Hs-578 Bst normal breast cells, WI38 and HDF human fibroblasts were obtained from the American Type Culture Collection (ATCC) and grown as recommended. Transformed rat fibroblast cell lines (BRN-1, -2, -4, -6 and -7) were derived from the transfection of rat embryo fibroblasts with polyoma large-T antigen and T24-H-*ras* (Engel *et al.*, 1993). These cells were maintained in Dulbecco's modified Eagle medium (DMEM) supplemented with 5% fetal calf serum, 2 mM glutamine, penicillin (100 units $ml^{-1}$) and streptomycin (100 $\mu g\ ml^{-1}$) (reagents from Flow Laboratories, Irvine, UK) at 37°C/7% carbon dioxide.

### *Tumour tissue samples*

Twenty malignant breast tumours (ten node-negative and ten node-positive) were analysed. In addition, we examined five non-cancerous breast lesions (three fibroadenomas, one hamartoma and one ductal hyperplasia).

Non-necrotic tumour tissue was processed for two-dimensional electrophoresis (2-DE) as previously described (Franzén *et al.*, 1993). Material from human breast lesions were collected immediately after resection. Tumours were cut and cells from a macroscopically viable area were collected by scraping with a scalpel and then were resuspended in ice-cold L15 medium supplemented with 5% calf serum. Scraping was found to preferentially detach tumour cells from the tissue (as revealed by staining of smears and sections). Samples were further enriched for tumour cells by removal of connective tissue by filtering of erythrocytes by centrifugation in Percoll. Serum proteins were removed by

Correspondence: B Franzén
Received 11 July 1995; revised 23 October 1995; accepted 1 November 1995

repeated washing in phosphate-buffered saline (PBS). All steps were performed on ice in the presence of protease inhibitors. Cells were lysed in a sodium dodecyl sulphate (SDS)-containing buffer, treated with DNAase and RNAase, and dissolved in sample buffer containing detergents (NP40 and CHAPS) as described (Franzén et al., 1993). Staining of cells extracted from carcinomas showed that these were usually >90% lesion-specific cells, free from stromal fibroblasts and other contaminating cells.

*Electrophoresis*

2-DE was performed by standard procedures as described (Anderson, 1988; Franzén et al., 1993). Resolyte (2%, pH 4–8, BDH) was used for isoelectric focusing, and 10% or 10–13% linear gradient SDS-polyacrylamide gels in the second dimension. Gels were stained with silver nitrate according to standard procedures.

*Identification of tm isoforms*

Tropomyosins were purified from WI38 fibroblasts as described by Matsumura and Yamashiro-Matsumura (1985). Purified protein was mixed with breast carcinoma (MDA-MB-231) extracts and subjected to 2-DE. In addition, 2-DE maps were prepared from WT2 embryonal rat fibroblasts and matched with the REF52 database (Garrels and Franza, 1989). In all analyses, tm isoforms were identified by matching the migration of protein with a reference pattern (Figure 1a–c) using PDQUEST software.

*Scanning and image analysis*

2-DE gels were scanned at 100 $\mu$m resolution using a PD laser densitometer from Molecular Dynamics. Data were analysed using the PDQUEST software (Garrels et al., 1984) (Pharmacia Biotech, Uppsala, Sweden). Background was subtracted, peaks located and quantitated. Tropomyosin 5 was used as standard for normalisation. The levels of tm 5 were found to parallel the levels of a number of other proteins expressed at similar levels (as their fraction of total integrated optical density in the gels) in 12 cases in which total 2-DE patterns were analysed. One of these proteins was identified as elongation factor 1$\beta$. It was found previously that tm 5 is insensitive to transformation and growth conditions (Garrels and Franza, 1989). We therefore chose to use tm 5 as an internal standard for quantitation (quantity of tm 5 = 100 units).

### Results

*Analysis of tm isoforms in human breast carcinoma tumours*

Tumour cells were extracted from non-cancerous human breast lesions and breast carcinoma tissue and prepared for 2-DE. Tm polypeptides were identified by co-electrophoresis of purified proteins and by matching with the REF52 database. Five tropomyosin isoforms were resolved (Figure 1) and the relative amounts of these polypetides were determined by scanning and quantified using PDQUEST software (Garrels et al., 1984). As shown in Figure 2 and Table I, the expression of the high molecular weight tms (tm 1, tm 2 and tm 3) was 4–5 fold higher in non-cancerous lesions compared with carcinomas. These differences were statistically significant for each protein at the level of $P<0.05$ (Mann–Whitney). Higher levels of tm 1 and tm 2 were observed in all three cases of fibroadenoma and in the single case of ductal hyperplasia compared with any of the cases of carcinoma (Table I and Figure 2). An intermediate level of tm 2 was observed in the single case of ductal hyperplasia, a

**Figure 1** Panel of the tm area from 2-DE gels representing; a reference pattern for PDQUEST analysis showing tm isoforms 1–5 (**a**), WI-38 fibroblasts, total proteins (**b**) and tm isoforms 1–5 purified from WI-38 fibroblasts (**c**). Non-cancerous breast lesions (**d–g**); fibroadenomas (**d–e**), hamartoma (**f**) and an intraductal hyperplasia (**g**). Node-negative (**h–k**) and node-positive carcinomas of the breast (**l–p**). Each 2-DE gel is shown with the acidic side on the left hand. Numbers indicate the appropriate tm isoform, when detectable (compare **c**). Circles indicate the expected position of tms.

**Figure 2** Histogram showing relative expression levels of tm isoforms 1–3 in non-cancerous (□) and cancerous (■) breast lesions. The average and standard deviation of five and 20 cases respectively is presented. Significantly lower levels of tms 1–3 were observed in breast carcinomas.

breast lesion that can be considered as a precancerous lesion. The expression of tm 3 was highly variable between carcinomas (3.5–71 units). In three carcinomas, the levels of tm 3 were found to be higher than the level observed in the ductal hyperplasia. The levels of tm 4 did not significantly differ between fibroadenomas and carcinomas (not shown). Tm 5 was used as an internal standard for quantitation (see Materials and methods).

The expression of tropomyosin isoforms was examined in seven human breast carcinoma cell lines, in breast derived

**Figure 3** Histogram showing relative expression levels of tropomyosin isoforms 1–3 in node-negative (□) and node-positive (■) breast carcinomas. The average and standard deviation of ten cases from each group is presented. A significantly higher level of tm 1 was observed in node-positive cases (*).

**Table I** Levels of high-$M_r$ tms in clinical breast material (relative integrated optical density, tm 5 = 100 units)

| | *tm 1* | *tm 2* | *tm 3* | |
|---|---|---|---|---|
| Breast carcinomas (*n* = 20) | 13 | 7.7 | 27 | (ductal, invasive) |
| | (0.9–27) | (0–14) | (3.5–71) | |
| Non-cancerous breast lesions (*n* = 5) | 62 | 39 | 103 | |
| Fibroadenomas (*n* = 3) | 54 | 39 | 121 | |
| Case 122 | 44 | 35 | 77 | (50% epithelial cells)[a] |
| Case 124 | 45 | 26 | 164 | (20% epithelial cells)[a] |
| Case 86 | 72 | 56 | 121 | (50% epithelial cells)[a] |
| Hamartoma | 80 | 59 | 105 | (50% epithelial cells)[a] |
| Ductal hyperplasia[b] (case 41) | 67 | 18 | 48 | (60% epithelial cells)[a] |

[a] Estimated from histological examination of stained sections. [b] Proliferative lesion with a combination of intraductal hyperplasia without atypia, sclerosing adenosis and fibrocystic disease.

**Table II** Levels of high-$M_r$ tms in breast-derived cell lines and in fibroblasts (relative integrated optical density, tm 5 = 100)

| *Cell line* | *tm 1* | *tm 2* | *tm 3* | |
|---|---|---|---|---|
| MCF-7 | 2.4 | 2.4 | 27 | |
| T47D | 2.0 | 4.0 | 41 | |
| BT 549 | 27 | 2.1 | 29 | |
| SK-BR-3 | 5.7 | 4.8 | 32 | |
| MDA-MB-134 | 0.7 | 0.7 | 5.3 | |
| MDA-MB-231 | 1.2 | 0.4 | 0.8 | |
| ZR-75-30 | 0.6 | 1.2 | 1.2 | |
| Mean values | 5.7 | 2.2 | 20 | |
| 578 Bst | 66 | 32 | 58 | (breast derived, non-tumorigenic, fibroblast-like) |
| WI38 | 80 | 17 | 38 | (fibroblast strain) |
| HDF | 76 | 22 | 21 | |

**Table III** Levels of high $M_r$ tms in 12V-H-*ras* transformed rat embryo fibroblasts

| *Cell strain* | *tm 1* | *tm 2* | *tm 3* | *Metastatic capacity*[a] |
|---|---|---|---|---|
| BRN-1 | 22 | 6.3 | 2.9 | + + + |
| BRN-7 | 17 | 4.5 | 2.1 | + + + |
| BRN-6 | 10 | 0.4 | 0.4 | + + |
| BRN-2 | 0.4 | 0.9 | 1.3 | + |
| BRN-4 | 0.4 | 1.5 | 0.5 | + |
| Embryo fibroblasts | 154 | 99 | 22 | |

[a] Data from Engel *et al.* (1993): + + +, all mice showed massive (> 30 nodes per section) lung metastases after i.v. injection of $2 \times 10^5$ cells; + +, all mice showed 10–30 metastases per section; + most mice show no or few (⩽ 10) colonies/section.

Hs-578 Bst cells and in human fibroblasts (Table II). Whereas tm 1 and tm 2 levels were similar in Hs-578 Bst cells and fibroblasts, they were lower in the breast carcinoma cell lines. Similar to the observations made in tumour tissue, tm 3 expression in cell lines varied considerably (0.8–41 units). Four of the cell lines showed higher levels of tm 3 than human diploid fibroblasts (HDFS).

### *Higher levels of tm 1 in node-positive compared with node-negative breast carcinomas*

Tm levels were compared in primary carcinomas from cases with or without lymph node metastases. As shown in Figure 3, tm 1 levels were 1.7-fold higher in node-positive compared with node-negative tumours. This difference was statistically significant at the level of $P<0.05$ (Mann–Whitney). No difference was observed in the levels of tm 2 or tm 3 between node-positive and -negative tumours.

### *Tm 1 expression in 12V-H-*ras transformed rat fibroblasts

Tropomyosin isoform expression was examined in rat fibroblasts and five 12V-H-*ras* transformed rat fibroblasts with varying capacities for experimental metastasis. A dramatic decrease in the expression of tm 1, tm 2 and tm 3 was observed in all transformed cell lines (Table III). Interestingly, higher levels of tm 1, 2 and 3 were observed in the most metastatic cell lines (BRN-1 and BRN-7). The differences in expression was most pronounced for tm 1. An intermediate level of tm 1 was observed in the moderately metastatic BRN-6 cell line.

### Discussion

Although alterations in tm isoform expression have been described in transformed cell lines in several previous studies, studies of tm expression in human tumour cells *in situ* are rare. In the present study, tumour cells were purified from both non-cancerous breast lesions (including benign tumours) and from breast carcinomas. Total cell extracts were then subjected to two-dimensional gel electrophoresis. By this procedure, we were able to obtain high-resolution polypeptide maps from tumour cells and were able to measure the total (insoluble and soluble) cellular quantitities of tms 1–5.

Our data clearly show that high $M_r$ tropomyosins are down-regulated in breast carcinoma. The levels of tms 1 and 2 were lower in all carcinomas than in any of the non-malignant tissues examined. Similarly, tm 3 was also lower in the carcinomas. In three cases, however, higher levels of tm 3 were observed in carcinomas compared with the case of ductal hyperplasia. We conclude from these results that the expression of high-$M_r$ tropomyosin isoforms is suppressed not only *in vitro* but also *in vivo*.

Previous studies of tms in transformed fibroblast and breast carcinoma cell lines have uniformly identified suppression of high $M_r$ tms (Hendricks and Weintraub, 1981; Bhattacharya *et al.*, 1988). We were able to reproduce these results using 12V-H-*ras* transformed rat embryo fibroblasts and breast carcinoma cell lines. Strong down-regulation of all three high-$M_r$ tropomyosin isoforms was observed in *ras*-transformed fibroblasts compared with untransformed cells. In a previous report, Garrels and Franza (1989) showed that tm 2 is suppressed to an intermediate level in morphologically SV40 transformed non-tumorigenic REF52 fibroblast clones (WT2 and WT6) as compared with tumorigenic clones. We have observed intermediate levels of tm 2 expression in WT2 cells compared with 12V-*ras* transformed rat embryo fibroblasts (our unpublished observations), confirming this finding. The levels of high-$M_r$ tropomyosins in *in vitro* cultured breast carcinoma cell lines were in the same range as those observed in tumours, but the average levels were lower. This could reflect some contamination in tumour samples, or may simply be a result of cell lines not being representative of primary tumour material, or that cell lines undergo modifications *in vitro* when they are established from primary cells.

Interestingly, the levels of tm 1 were found to be elevated in primary tumours that had given rise to lymph node metastases. Tm 1 expression was higher in two strongly metastatic 12V-*ras* transformed cell lines compared with three weakly metastatic lines. This difference was most pronounced for tm 1. In a previous study (Okuzawa *et al.*, 1994), we found tm 1 expression in four out of four small-cell lung carcinomas, but did not detect tm 1 in six out of eight non-small-cell lung carcinomas. Small-cell lung carcinomas are known to be highly metastatic, whereas non-small-cell lung carcinomas are not. These findings raise the possibility that higher levels of tm 1 may induce cellular motility or affect other functions that may contribute to metastasis.

Because of the promising possibility of a future use of tm markers in the field of tumour diagnosis and malignancy grading, we plan to extend the investigation by examining additional clinical material representing different stages of malignant transformation and tumour progression.

**Acknowledgements**
We thank Drs Martin Bäckdahl and Göran Wallin at the Department of Surgery and Eva Edholm at the mammography unit for the supply of clinical material, and Elina Ericsson at the Department of Pathology for histopathological examinations. This work was supported by Cancerföreningen i Stockholm and Cancerfonden.

### References

ANDERSON NL. (1991). *Two-Dimensional Electrophoresis, Operation of the ISO-DALT System*, 2nd edn, pp. 17–97. Large Scale Biology press: Rockville, MD.

BHATTACHARYA B, CIARDIELLO F, SALOMON DS AND COOPER HL. (1988). Disordered metabolism of microfilament proteins, tropomyosin and actin, in mouse mammary epithelial cells expressing the Ha-*RAS* oncogene. *Oncogene Res.*, **3,** 51–65.

BHATTACHARYA B, PRASAD GL, VALVERIUS EM, SALOMON DS AND COOPER HL. ((1990). Tropomyosin of human mammary epithelial cells: consistent defects of expression in mammary carcinoma cell lines. *Cancer Res.*, **50,** 2105–2112.

COOPER HL, BHATTACHARYA B, BASSIN RH AND SALOMON DS. (1987). Suppression of synthesis and utilization of tropomyosin in mouse and rat fibroblasts by transforming growth factor alpha: a pathway in oncogene action. *Cancer Res.*, **47**, 4493–4500.

ENGEL G, POPOWICZ P, MARSHALL H, FRANZÉN B, OKUZAWA K, AUER G AND LINDER S. (1993). Limited invasive capacity of *plt+ras* transformed rat fibrosarcoma cells effective in experimental metastasis. *Int. J. Oncology*, **3**, 457–465.

FRANZÉN B, OKUZAWA K, LINDER S, KATO H AND AUER G. (1993). Non-enzymatic extraction of cells from clinical tumour material for analysis of gene expression by two-dimensional gel electrophoresis. *Electrophoresis*, **14**, 382–390.

GARRELS JI AND FRANZA BJ. (1989). Transformation-sensitive and growth-related changes of protein synthesis in REF52 cells. A two-dimensional gel analysis of SV40-, adenovirus-, and Kirsten murine sarcoma virus-transformed rat cells using the REF52 protein database. *J. Biol. Chem.*, **264**, 5299–5312.

GARRELS JI, FARRAR JT AND BURWELL CB. (1984). The QUEST system for computer-analyzed two-dimensional gel electrophoresis of proteins. In Celis JE and Bravo R. (eds.) *Two-dimensional Gel Electrophoresis of Proteins: Methods and Applications*. Academic Press: New York.

GLUCK UD, KWIATKOWSKI J AND BEN-ZE'EV A. (1993). Suppression of tumorigenicity of simian virus 40-transformed 3T3 cells transfected with α-actinin cDNA. *Proc. Natl Acad. Sci. USA*, **90**, 383–387.

HENDRICKS M AND WEINTRAUB H. (1981). Tropomyosin is decreased in transformed cells. *Proc. Natl Acad. Sci. USA*, **78**, 5633–5637.

LEES-MILLER JP AND HELFMAN DM. (1991). The molecular basis for tropomyosin isoform diversity. *Bio Essays*, **13**, 429–437.

MATSUMURA F AND YAMASHIRO-MATSUMURA S. (1985). Purification and characterization of multiple isoforms of tropomyosin from rat cultured cells. *J. Biol. Chem.*, **260**, 13851–13859.

OKUZAWA K, FRANZÉN B, LINDHOLM J, LINDER S, HIRANO T, BERGMAN T, EBIHARA Y, KATO H AND AUER G. (1994). Characterization of gene expression in clinical lung cancer materials by two-dimensional polyacrylamide gel electrophoresis. *Electrophoresis*, **15**, 382–390.

PRASAD GL, FULDNER RA AND COOPER HL. (1993). Expression of transduced tropomyosin 1 cDNA suppresses neoplastic growth of cells transformed by the ras oncogene. *Proc. Natl Acad. Sci. USA*, **90**, 7039–7043.

RAZ A AND GEIGER B. (1982). Altered organization of cell-substrate contacts and membrane associated cytoskeleton in tumor cell variants exhibiting different metastatic capabilities. *Cancer Res.*, **42**, 5183–5190.

TAKENAGA K AND MASUDA A. (1994). Restoration of microfilament bundle organization in v-*raf*-transformed NRK cells after transduction with tropomyosin 2 cDNA. *Cancer Lett*, **87**, 47–53.

VANDERKERCKHOVE J, BAUW GK, VANCOMPERNOLLE B, HONORE B AND CELIS J. (1990). Comparative two dimensional gel analysis and microsequencing identifies gelsolin as one of the most prominent downregulated markers of transformed human fibroblasts and epithelial cells. *J. Cell. Biol.*, **111**, 95–102.